# Radiation Dosimetry of Theragnostic Pairs for Isotopes of Iodine in IAZA

**DOI:** 10.3390/pharmaceutics14081655

**Published:** 2022-08-09

**Authors:** Hans-S. Jans, Daria Stypinski, Piyush Kumar, John R. Mercer, Stephen A. McQuarrie, Alexander J. B. McEwan, Leonard I. Wiebe

**Affiliations:** 1 Department of Oncology, University of Alberta, Edmonton, AB T6G 1Z2, Canada; 2 Pfizer Inc., New York, NY 10017, USA; 3 Faculty of Pharmacy and Pharmaceutical Sciences, University of Alberta, Edmonton, AB T6G 2R3, Canada; 4 Ariceum Therapeutics GmbH, 13125 Berlin, Germany

**Keywords:** theragnostics, theranostics, dosimetry, pharmacokinetics, iodine, IAZA, ^131^I, ^124^I, ^123^I, radionuclide, MIRD

## Abstract

Theragnostic pairs of isotopes are used to infer radiation dosimetry for a therapeutic radiopharmaceutical from a diagnostic imaging study with the same tracer molecule labelled with an isotope better suited for the imaging task. We describe the transfer of radiation dosimetry from the diagnostic radioiodine isotope ^123^I, labelled for the hypoxia tracer molecule iodoazomycin arabinoside ([^123^I]IAZA), to isotopes ^131^I (therapeutic) and ^124^I (PET imaging). Uncertainties introduced by the dissimilar isotope half-lives are discussed in detail. Radioisotope dosimetries for [^123^I]IAZA were obtained previously. These data are used here to calculate residence times for ^131^I and ^124^I and their uncertainties. We distinguish two cases when extrapolating to infinity: purely physical decay (case A) and physical decay plus biological washout (case B). Organ doses were calculated using the MIRD schema with the OLIDNA/EXM code. Significant increases in some organ doses (in mSv per injected activity) were found for ^131^I and ^124^I. The most affected organs were the intestinal walls, thyroid, and urinary bladder wall. Uncertainty remained similar to ^123^I for case A but considerably greater for case B, especially for long biological half-lives (GI tract). Normal tissue dosimetries for IAZA must be considered carefully when substituting isotope species. A long biological half-life can significantly increase dosimetric uncertainties. These findings are relevant when considering PET imaging studies with [^124^I]IAZA or therapeutic administration of [^131^I]IAZA.

## 1. Introduction

Personalized radiation dosimetry in nuclear medicine refers to the individual prescription of radiopharmaceuticals. New developments in the field of targeted radionuclide therapy (TRT) combine a diagnostic probe with a matched therapeutic agent [1,2,3]. A previous dosimetry study can then inform both the therapeutic prescription and the benefit-risk assessment of the therapeutic radiopharmaceutical.

Iodine isotopes were first used for both diagnostics and radioisotope therapy [4]. ^123^I or ^124^I are used diagnostically because of their low emission energy for scintigraphic imaging or their positron emission for PET imaging, respectively. Because of its availability and lower cost, ^131^I has been used for diagnostic imaging, but its electron emission energies and half-life also make it a potent therapy agent [3], as summarized in Table 1.

A theragnostic pair of radiopharmaceuticals can incorporate isotopes of the same element, leaving the molecular structure unaltered. However, sufficiently similar biodistribution can also result with different pairs of elements, e.g., ^68^Ga/^177^Lu-Dotatate [1]. It is assumed that its biokinetics essentially remains unaltered between the diagnostic study and therapy. Radiation dosimetry is revealed by a series of diagnostic studies and subsequently transferred to the therapeutic case by accounting for differences in physical half-life and isotope emissions.

**Figure 1:**
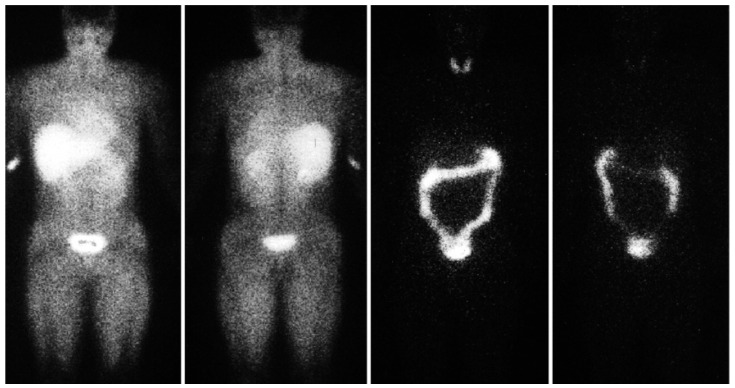
Typical immediate (0–30 min) anterior (**left**) and posterior (**left centre**) views and 22 h anterior (**right centre**) and posterior (**right**) planar images after ^123^I-IAZA intravenous administration to volunteers. Images were adapted from Stypinski et al. 2001. [9].

Hypoxia is considered to play a major role in solid cancer growth, metastasis and resistance to treatment; a number of approaches have been developed to address these challenges [6]. The iodine-labelled radiotracer iodoazomycin arabinoside (IAZA) is a first-generation hypoxia imaging agent that binds selectively to hypoxic sites in tumours and other tissues [7]. Image-based pharmacokinetic and radiation dosimetric data for [^123^I]IAZA have been reported for healthy, exercising, adult volunteers [8] (Figure 1). Data from the diagnostic imaging study of sedentary healthy volunteers supported a classical two-compartment pharmacokinetic model derived from sequential venous blood and urine samples. SPECT region of interest (ROI) image analyses provided comparable total body mean residence times and urinary clearance estimates. ROI data from serial images were used to generate time-activity curves for individual organs to derive detailed radiation dosimetry estimates for ^123^IAZA [9].

Motivated by a renewed interest in radioisotope molecular theragnostics, data from the latter study have now been re-evaluated to derive radiation dosimetry for IAZA labelled with ^124^I for diagnostic PET imaging and for ^131^I as the therapeutic isotope. Absorbed organ doses for theragnostic pairings with ^131^I for the targeted treatment of hypoxic malignancies are presented. Furthermore, the uncertainty inherent in this dosimetric transfer is quantified both with and without considering the biological washout of IAZA. The dose conversion formalism and considerations for extrapolation towards infinite time points are discussed in a generally applicable manner, as are implications for dosimetric uncertainty.

## 2. Materials and Methods

Dosimetric imaging studies of the hypoxia tracer [^123^I]IAZA in six healthy volunteers, who had been administered Lugol’s solution, had been carried out previously [9]. Dual-head, whole-body gamma camera scans had been acquired at five time points $t_{i}$ of 0.5, 1–2, 3–4, 6–8, and 20–24 h post-injection (p.i.). Tracer uptakes were discernable in the thyroid, liver, kidneys, gastrointestinal (GI) tract, and whole-body (WB), which were designated source organs. Regions-of-interest (ROIs) were drawn around source organs and their activity derived, as well as for “all body” (AB), the whole-body for less the urinary bladder (UB). Time-activity curves (TACs), $A\left(t_{i}\right)\equiv A_{i}$, were generated for each source organ of each volunteer.

To obtain cumulated activity, $\tilde{A}$ (i.e., the total number of radioactive decays in a given source organ), the measured TACs were integrated numerically up to the last measured time point (20–24 h p.i.). In addition, cumulated activity beyond the last measured time point can contribute a significant dose and needs to be taken into account. It was determined by extrapolation to infinity using two different methods: by assuming only the physical decay of the isotope (case A) and by additionally accounting for biological washouts from the source organ (case B).

For case A, the number of decays occurring beyond $t_{5}$, ${\tilde{A}}_{5+}$, was simply calculated by integrating physical decay between $t_{5}$ and infinity, yielding:

(1) $${\tilde{A}}_{5+}=A_{5}/\lambda_{p}$$

where $A_{5}$ denotes any activity at the 5th time point and $\lambda_{p}=\ln\left(2\right)/t_{1/2,p}$ is the physical decay constant.

Case B includes the effect of biological washout for times $t>t_{5}$ and was modeled by mono-exponential fit to $A_{4}$ and $A_{5}$:

(2) $$A\left(t_{i}\right)=A_{0}^{\prime }\;e^{-\left(\lambda_{p}+\lambda_{b}\right)t_{i}}\;\;\;⟺\;\;\;\lambda_{b}+\lambda_{p}=\frac{\ln\left(A_{4}/A_{5}\right)}{t_{5}-t_{4}}$$

where $\lambda_{b}=\ln\left(2\right)/t_{1/2,b}$ is the biological decay constant. ${\tilde{A}}_{5+}$ for case B was calculated similarly to case A but by replacing $\lambda_{p}$ with $\lambda_{p}+\lambda_{b}$ in Equation (1).

**Figure 2:**
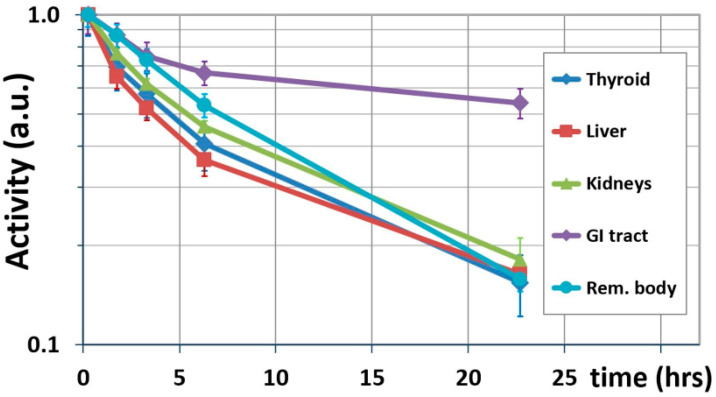
Biological washout of IAZA from source organs (mean from 6 healthy volunteers). Washout decreases activity to below 20% for all organs by ~21 h p.i. except the GI tract, where the last time point is at 54% of the initial activity.

To obtain the TACs for either ^131^I or ^124^I, the effect of physical decay of ^123^I was removed from the measured TACs by multiplying with a factor $e^{\lambda_{p}^{I-123}t_{i}}$:

(3) $$A_{i,b}=A_{i}e^{\lambda_{p}^{I-123}t_{i}}$$

where $\lambda_{p}^{I-123}$ is the physical decay constant for ^123^I. Figure 2 shows the results of this interim step, showing biological washouts only. Table 2 lists the terminal biological half-lives calculated for source organs using Equation (2).

The physical half-lives of ^124^I and ^131^I were then introduced by weighting each data point with a factor $e^{-\lambda_{p}^{X}t_{i}}$. Each originally measured time point was, thus, multiplied by factor:

(4) $$f^{X}\left(t_{i}\right)=e^{\left(\lambda_{p}^{I-123}-\lambda_{p}^{X}\right)t_{i}}$$

where X denotes either ^131^I or ^124^I; thus, the following is obtained:

(5) $$A_{i}^{X}=A_{i}^{I-123}\;f^{X}\left(t_{i}\right)$$

Cumulated activity ${\tilde{A}}^{X}$ was obtained as for ^123^I as the sum of the are under the TAC and interpolation to infinity assuming either physical decay only (case A) or physical decay and biological washout (case B) for each source organ S:

(6) $${\tilde{A}}_{S}^{X}={\tilde{A}}_{5,S}^{X}+{\tilde{A}}_{5+,S}^{X}$$

The number of decays per injected activity, τ (also known as residence time), was obtained by normalizing to the injected activity, $A_{inj}^{X}$:

(7) $$\tau_{S}^{X}=\frac{{\tilde{A}}_{S}^{X}}{A_{inj}^{X}}$$

Organ doses were computed using the MIRD formalism [10,11] as implemented in the OLINDA/EXM 1.1 code (Vanderbilt 2007) [12].

Obtaining τ from the images’ ROIs was straightforward for the thyroid, liver, and kidneys. Residence times for bladder content and sub-regions of the GI tract utilized their respective models [13,14] as implemented in the OLINDA/EXM code. Following the methodology employed by Stypinski et al. [9], the bladder model was employed by first calculating the fraction entering the urinary bladder (UB) as follows: $f_{UB}^{X}=1-f_{GI}^{X}$, where $f_{GI}^{X}$ is the fraction of decays occurring in the GI tract relative to the all body (AB) region, $f_{GI}^{X}=\tau_{GI}^{X}/\tau_{AB}^{X}$ (2 h voiding interval). The ICRP 30 GI model was used by entering the fraction $f_{GI}^{X}$ into the respective OLINDA module, which then populated the residence times for the small intestine (SI) and upper and lower large intestine (ULI and LLI). Lastly, we determined the number of decays in the “remainder body” (RB) by subtracting the decays in all other source organs from those in the whole-body region. Residence times are summarized in Table 3 for both cases A and B. The averages determined from dose calculations performed with data from each of the six volunteers are shown.

Dosimetric uncertainty is introduced due to the imprecision of each measured activity $A_{i}$ [15,16]. Here, we additionally consider the uncertainty caused by the short half-life of ^123^I, which necessitates terminating measurements after 24 h. Assumptions have to be made, therefore, about the biokinetic excretion of longer-lived radiopharmaceuticals (here, ^124^I and ^131^I) beyond the last measured time point, $t_{5}$. We discuss two different assumptions and the uncertainties arising from each: either the radiopharmaceutical is irreversibly bound to tissue (no biological washout, case A) or biological washout and physical decay (case B).

If the radiopharmaceutical is irreversibly bound for $t>t_{5}$ (case A), the cumulated activity ${\tilde{A}}_{5+}$ is given by Equation (1). Since $\lambda_{p}$ is known, the uncertainty, $\delta {\tilde{A}}_{5+}$, is determined by the measurement uncertainty for $A_{5}$ such that $\delta {\tilde{A}}_{5+}/{\tilde{A}}_{5+}=\delta A_{5}/A_{5}$. Using error propagations and assuming the mutual independence of ${\tilde{A}}_{5+}$ and ${\tilde{A}}_{5}$, the uncertainty of the total cumulated activity, $\delta \tilde{A}$, becomes:

(8) $${\left(\delta \tilde{A}\right)}^{2}={\left(\delta \left({\tilde{A}}_{5}+{\tilde{A}}_{5+}\right)\right)}^{2}={\left(\delta {\tilde{A}}_{5}\right)}^{2}+{\left(\delta {\tilde{A}}_{5+}\right)}^{2}$$

$\delta {\tilde{A}}_{5}$ is caused by uncertainties in measured activities $A_{i}$. Following the approach taken in [15] leads to $\delta {\tilde{A}}_{5}/{\tilde{A}}_{5}=\delta A/A$, where δA/A is a representative, relative uncertainty for activity measurements at individual time points. The relative uncertainty of the total cumulated activity then becomes:

(9) $$\frac{\delta \tilde{A}}{\tilde{A}}=\frac{1}{\tilde{A}}\sqrt{{\left(\delta {\tilde{A}}_{5}\right)}^{2}+{\left(\delta {\tilde{A}}_{5+}\right)}^{2}}=\frac{1}{\tilde{A}}\sqrt{{\left({\tilde{A}}_{5}\frac{\delta A}{A}\right)}^{2}+{\left({\tilde{A}}_{5+}\frac{\delta A_{5}}{A_{5}}\right)}^{2}}=\frac{\delta A}{A}\frac{\sqrt{{\tilde{A}}_{5}^{2}+{\tilde{A}}_{5+}^{2}}}{\left({\tilde{A}}_{5}+{\tilde{A}}_{5+}\right)}<\frac{\delta A}{A}$$

where we replaced the ratio $\delta A_{5}/A_{5}$ by a representative uncertainty δA/A. The relative uncertainty in total cumulated activity is, therefore, lower than the error in the individual activity measurements in this case. The values of the relative uncertainty δA/A are not exactly known from Stypinski et al.’s work but are greater than 10% [9]. Here, we will assume δA/A=20%.

In the second scenario (case B), biological half-lives (Table 2) are included in calculations when determining ${\tilde{A}}_{5+}$. We assume that the last two measured activities, $A_{4}$ and $A_{5}$, define the terminal mono-exponential elimination phase. ${\tilde{A}}_{5+}$ then follows from Equations (1) and (2), and its uncertainty is caused by uncertainties in $A_{4}$ and $A_{5}$. Here, error propagation (Appendix A) leads to:

(10) $$\frac{\delta {\tilde{A}}_{5+}}{{\tilde{A}}_{5+}}=\frac{\delta A_{5}}{A_{5}}\;\cdot \;\eta \left(\frac{A_{4}}{A_{5}}\right)$$

η is a function of the activity ratio $A_{4}/A_{5}$; values of 3 and 1.5 for $A_{4}/A_{5}$, for example, cause the uncertainty $\delta {\tilde{A}}_{5+}/{\tilde{A}}_{5+}$ to be a factor η of 1.9- and 3.8-times greater than the measurement uncertainty of $\delta A_{5}/A_{5}$. Source organs with a rapid clearance (i.e., large ratio $A_{4}/A_{5}$) will exhibit less uncertainty in the extrapolated activity; this is intuitively clear because a ratio of $A_{4}/A_{5}=1$ would mean infinite extrapolated cumulated activity with no ability to ascertain its uncertainty. As before (Equation (9)), the uncertainty in ${\tilde{A}}_{5+}$ contributes to the uncertainty in $\tilde{A}$; its relative uncertainty now becomes the following:

(11) $$\frac{\delta \tilde{A}}{\tilde{A}}=\frac{1}{\tilde{A}}\sqrt{{\left(\delta {\tilde{A}}_{5}\right)}^{2}+{\left(\delta {\tilde{A}}_{5+}\right)}^{2}}=\frac{1}{\tilde{A}}\sqrt{{\left({\tilde{A}}_{5}\frac{\delta A}{A}\right)}^{2}+{\left({\tilde{A}}_{5+}\frac{\delta A_{5}}{A_{5}}\eta \right)}^{2}}=\frac{\delta A}{A}\frac{\sqrt{{\tilde{A}}_{5}^{2}+{\tilde{A}}_{5+}^{2}\eta^{2}}}{\left({\tilde{A}}_{5}+{\tilde{A}}_{5+}\right)}$$

We note that the inequality present in Equation (9) now does not hold because η>1.

## 3. Results

**Figure 3:**
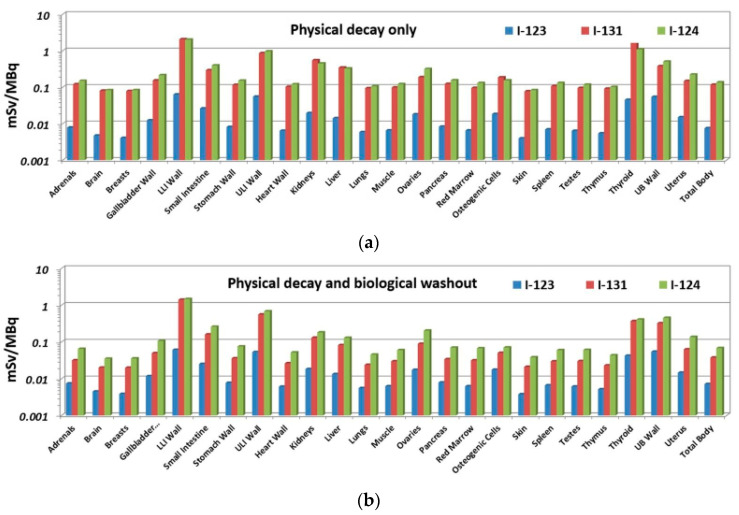
Organ doses (in mSv per injected dose) for the three isotopes of iodine and the two cases of elimination beyond the last measured time point: (**a**) physical decay only (top) and (**b**) physical decay and biological washout (bottom).

Calculated organ doses are listed in Table 4 for both cases: physical decay only (case A) and physical decay plus biological elimination (case B). Figure 3 shows the data in graphical form. Re-calculated values for ^123^I deviate <10% on average from Stypinski et al. [8], with the exception of osteogenic cells, in which greater differences result from modified absorbed fractions introduced in OLINDA [12].

Doses for both ^124^I and ^131^I are found to be significantly greater compared to ^123^I. When physical decay is only considered (case A), the greatest dose increase is predicted for the LLI wall (×32 for both ^131^I and ^124^I), thyroid (×37 and ×24 for I-31 and ^124^I, respectively), kidneys (×28 and ×23 for ^131^I and ^124^I, respectively), and liver (×24 and ×23 for ^131^I and ^124^I, respectively). Doses for ^131^I and ^124^I are predicted to be greater than 2 mSv/MBq for the LLI wall and more than 1 mSv/MBq for the thyroid; doses relative to the ULI wall approaches 1 mSv/MBq for both ^131^I and ^124^I. Effective doses are predicted to increase relative to ^123^I by a factor of about 22 for both ^131^I and ^124^I to almost 0.5 mSv/MBq.

If a biological washout is assumed to continue beyond the last measured time point (case B), dose estimates are significantly reduced relative to case A, owing to the more rapid washout from the “remainder body” (RB) and most other source organs. Using biological half-lives, per Table 2, decreases organ doses on average by 5% for ^123^I and by factors of 3.2 and 2.0 for ^131^I and ^124^I, respectively, relative to case A because biological washouts mostly affect the dosimetry of physically long-lived radioisotopes. For all isotopes, this decrease is the lowest for the GI tract because of its slowest biological washout.

Compared to case A, the inclusion of biologic washout in case B substantially reduces the predicted dose increase for the thyroid to less than a factor of 10 relative to ^123^I (×8.7 and ×9.7 for ^131^I and ^124^I, respectively). The relatively fast biological washout from this organ (Figure 2) is most likely due to the administration of Lugol’s solution prior to the administration of IAZA, which blocks the uptake of free radioiodine.

The dose for the LLI wall is also reduced for case B but remains greater than 1.4 mSv/MBq for both ^131^I and ^124^I, which is a factor of 23 above that for ^123^I. Other organs predicted in case B to receive a dose substantially greater than 0.1 mSv/MBq are the ULI wall (0.6 and 0.7 mSv/MBq for ^131^I and ^124^I, respectively), thyroid (0.4 mSv/MBq), and the UB wall (0.3 and 0.5 mSv/MBq for ^131^I and ^124^I, respectively). Absolute doses for kidneys and liver are predicted to be substantially reduced, with values for case B at 0.13 and 0.18 mSv/MBq and 0.08 and 0.13 mSv/MBq for ^131^I and ^124^I, respectively. Effective doses for case B are 2.4 and 3.0 mSv/MBq for ^131^I and ^124^I, respectively—an increase relative to ^123^I but almost halved compared to case A.

The relative uncertainties $\delta \tilde{A}/\tilde{A}$ (Equations (9) and (10)) are listed in Table 5.

## 4. Discussion

The elevated doses predicted here for IAZA radiolabelled with ^131^I or ^124^I compared to ^123^I (for identical administered activities) are caused by the longer physical half-life and the increased energy deposition per nuclear decay (Table 1).

The local (short-range electronic) energy deposition accounts for a factor ~7 increased self-dose of a source organ relative to ^123^I. The half-lives of ^131^I and ^124^I are longer than that of ^123^I by a factor of ~14.6 and ~7.6, respectively. These result in substantially elevated doses only if they are not dominated by biological washout. Doses from ^124^I are generally greater than for ^131^I (Figure 3) when the biological half-life is considered (case B); however, due to its longer physical half-life, doses from ^131^I will dominate those from ^124^I only if the physical half-life is considered beyond $t_{5}$ (case A), as is the case, e.g., for the LLI wall and thyroid.

As expected, the absorbed doses for ^124/131^I for case A are greater than for case B, an increase that is determined by both the isotopes’ half-lives and emission spectra. For $t>t_{5}$, the increase is quantified by the product of emission energies and half-lives, which yields factors of 14.6×7.2=105 for ^131^I and 7.6×7.2=55 for ^124^I (values from Table 1, accounting for electronic emissions only). The increase relative to ^123^I over all time points is, however, lower than these factors because the measured portions of the TACs, up to $t_{5}$, include biological washout. Only the increase in emission energy fully impacts the deposited dose at all times, while longer half-lives only partially impact the dose up to $t_{5}$. These combined effects cause the overall significant dose to increase for ^131^I and ^124^I (Table 4).

Photonic emissions deposit dose throughout the body. Both ^131^I and ^124^I emit more photon energy per nuclear decay than ^123^I (Table 1), causing greater doses in distant organs. Although chief photon emission energies are broadly similar for ^131^I and ^124^I, the latter emits approximately three-times greater total photon energy per decay, which is reflected in higher dose values for distant organs (Table 4).

The clinical question at hand will determine which of the two scenarios described here should be applied. Assuming physical decay only (case A) estimates the dosimetric ‘worst case’ for normal tissues and is an important consideration for radiation safety risk assessment and regulatory submission of novel radiotracers. Conversely, case A constitutes the ‘best case’ (most optimistic) scenario for a therapeutic dose delivered to malignancies in theragnostics. Here, it can be prudent to consider biological washout of the unbound radiopharmaceutical to obtain a more realistic dose value for the treatment target.

The uncertainty incurred by extrapolating dosimetry for long-lived isotopes from shorter-lived ones is an important consideration. We present here a quantitative uncertainty analysis for cumulated activity, expressed as $\delta \tilde{A}/\tilde{A}$ (Table 5). This uncertainty propagates to that of the residence time $\tau =\tilde{A}/A_{inj}$, which in turn propagates to uncertainty in absorbed dose D because $D/A_{inj}=S\cdot \tau$, where S is the S-value (a.k.a. ‘dose factor’) [9,11]. This is the case for source organs, for which cumulated activity has been directly measured and dose contributions from other organs are here considered negligible due to the greatly reduced S-values. Distant organs receive radiation doses from several source organs; their uncertainties are a combination of those of each source organ. Uncertainties in $A_{inj}$ and S will also be contributions. This work, however, is considering specifically the uncertainty caused by the transfer from ^123^I to ^131^I and ^124^I, which is quantified as $\delta \tilde{A}/\tilde{A}$.

**Figure A1:**
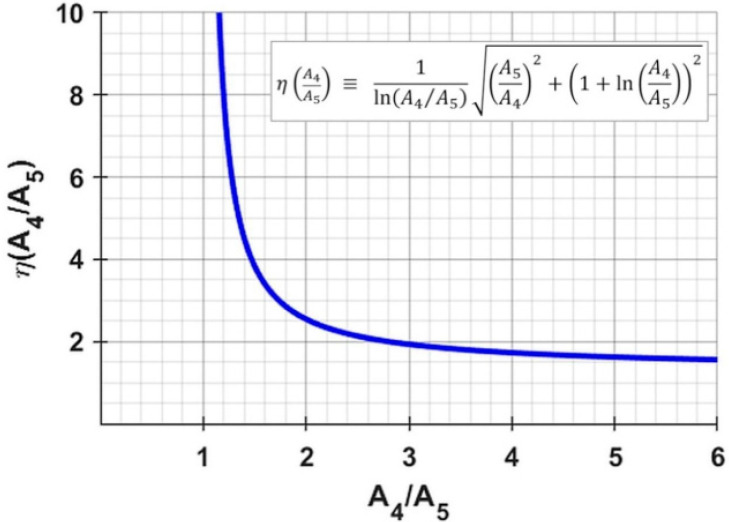
The function $\eta \left(A_{4}/A_{5}\right)$ as defined by Equation (A4).

When considering physical decay only (case A), all uncertainties $\delta \tilde{A}/\tilde{A}$ are smaller than the uncertainty assumed here for activity measurements (δA/A=20%), as per Equation (9). Similarly, small uncertainties are found for case B (biological washout), except for the GI tract. Figure 2 reveals that the GI tract has the longest biological half-life (57.5 h), resulting in the ratios $A_{4}/A_{5}$ being close to unity: 1.31 and 1.38 for ^131^I and ^124^I, respectively, resulting in large values for η of 5.5 and 4.7 (Figure A1); these result in high uncertainties in case B (Equation (11)) for the GI tract of 76.5% and 60.1% for ^131^I and ^124^I, respectively (Table 5). For case B, therefore, special attention needs to be directed to source organs with a long biological half-life. This underlines the importance of accurate activity measurements in order to minimize dosimetric uncertainty, especially if the biological washout is to be extrapolated to infinity.

Additional uncertainty is introduced by the choice of time points used to calculate biological half-life. Equation (2) assumes that $t_{4}$ and $t_{5}$ define the terminal, mono-exponential elimination phase. This would ideally be confirmed with a third data point located on the same curve. Unfortunately, acquiring a data point in between $t_{4}$ and $t_{5}$ was not possible due to logistical limitations (the scan would have had to take place during the night). The TAC values at the next-earlier time point, $t_{3}$, on the other hand, are located slightly above the line connecting $t_{4}$ and $t_{5}$ (Figure 2), suggesting that the distribution phase of IAZA is not completed at $t_{3}$. If the activities measured at $t_{4}$ were partially affected by the distribution phase (and therefore relatively elevated), the calculated terminal half-lives and absorbed doses would be under-estimated. Because of these practical limitations, the dose values found for case B represent lower limits.

## 5. Conclusions

We have transferred dosimetry for the theragnostic candidate radiopharmaceutical [^123^I]IAZA to the longer-lived isotopes ^131^I and ^124^I. As expected, higher absorbed doses are predicted for all organs, owing to greater S-values and longer physical half-life of ^131^I and ^124^I. Dosimetry estimates range depending on the approach, illustrating the importance of a full understanding of the biological behaviour of the radiopharmaceuticals for accurate individual patient dosimetry, especially in the theragnostic context [17,18,19].

The most conservative case, considering physical decay only (case A), leads to the greatest predicted dose increase for the LLI wall and thyroid, followed by kidneys and liver. A more physiologically realistic approach includes biological washout (case B) and results in significantly lower predicted doses for all organs except the GI tract, owing to its long biological half-life. Dosimetric uncertainties remain similar to those for ^123^I for case A, whereas they can significantly increase for case B, especially for organs with a long biological half-life (here the GI tract). Accurate activity measurement during the dosimetric study is particularly important in this case. The analysis presented here informs the benefit-risk assessment for the diagnostic or therapeutic use of [^124^I]IAZA or [^131^I]IAZA, respectively.

## Figures and Tables

**Table 1:** Pertinent iodine radioisotope properties [5].

Isotope	Half Life		Emissions			
			Electronic		Photonic	
	h	rel. to ^123^I	keV per Decay	rel. to ^123^I	keV per Decay	rel. to ^123^I
^123^I	13.2	1.0	26.7	1.0	170.1	1.0
^131^I	192.6	14.6	190.7	7.1	368.1	2.2
^124^I	100.2	7.6	193.4	7.2	1117.9	6.6

**Table 2:** Terminal biological half-life of source organs determined by exponential fit to the last two data points ($t_{4}$ and $t_{5}$ ) in Figure 2.

Source Organ	Biological Half-Life (Hours)
Thyroid	11.4
Liver	16.8
Kidney	15.0
GI tract	57.5
Rem. Body	7.3

**Table 3:** Residence times (RT), averaged over all six volunteers, in hours.

All Values in Units (Hours)	Physical Decay Only (Case A)			With Biological Washout (Case B)		
Source Organ	^123^I	^131^I	^124^I	^123^I	^131^I	^124^I
Kidneys	0.163	1.16	0.653	0.152	0.261	0.247
Liver	0.537	3.40	2.29	0.502	0.899	0.844
Thyroid	0.042	0.282	0.160	0.039	0.063	0.060
LLI Contents *	0.670	4.57	3.71	0.646	3.199	2.76
SI Contents *	0.423	0.866	0.786	0.407	0.606	0.584
ULI Contents *	0.818	2.69	2.34	0.788	1.88	1.74
UB Contents **	0.647	0.814	0.805	0.651	0.881	0.856
Rem. Body	6.26	32.4	19.4	5.96	8.10	8.21

* Determined from OLINDA’s GI model; ** determined from OLINDA’s voiding bladder model.

**Table 4:** Equivalent doses in µSv/MBq.

	Physical Decay Only (Case A)			Physical Decay and Biological Washout (Case B)		
Target Organ	^123^I	^131^I	^124^I	^123^I	^131^I	^124^I
Adrenals	7.8	120.8	149.2	7.4	31.7	64.7
Brain	4.7	80.1	82.6	4.5	20.0	34.9
Breasts	4.0	78.4	83.0	3.9	19.9	35.6
Gallbladder Wall	12.3	153.8	213.7	11.7	49.4	106.6
LLI Wall	63.1	2065.0	2010.0	60.8	1405.3	1464.2
Small Intestine	26.1	292.0	395.7	25.1	159.8	258.5
Stomach Wall	8.1	115.9	151.0	7.7	36.0	75.1
ULI Wall	55.0	859.2	962.8	52.9	557.0	680.0
Heart Wall	6.4	103.0	120.1	6.1	26.2	51.5
Kidneys	19.5	548.7	446.2	18.3	130.0	181.0
Liver	14.1	345.5	325.5	13.3	81.9	129.0
Lungs	5.9	94.0	105.8	5.6	23.8	45.1
Muscle	6.5	98.5	120.9	6.2	29.8	59.7
Ovaries	18.1	187.8	316.0	17.4	88.8	202.7
Pancreas	8.3	123.2	154.3	7.9	34.0	69.9
Red Marrow	6.5	95.9	130.2	6.2	31.5	67.1
Osteogenic Cells	18.4	186.0	154.0	17.6	50.4	70.5
Skin	4.0	77.2	82.9	3.8	21.0	38.2
Spleen	6.9	107.4	130.8	6.6	29.6	59.6
Testes	6.4	95.2	117.1	6.1	30.1	60.5
Thymus	5.4	91.3	101.7	5.2	23.0	43.3
Thyroid	45.1	1649.3	1082.3	42.1	367.3	406.3
UB Wall	54.4	377.5	502.2	54.3	322.5	451.3
Uterus	15.0	148.2	221.8	14.6	62.9	136.5
Total Body	7.5	116.6	135.5	7.2	37.5	67.9
Effective Dose	20.5	459.5	466.7	19.8	243.0	298.7

**Table 5:** Relative uncertainty of cumulated activities; the representative uncertainty for measuring activity, $\delta \tilde{A}/\tilde{A}$ , was assumed to be 20%.

	Physical Decay Only			With Biological Washout		
Source Organ	^123^I	^131^I	^124^I	^123^I	^131^I	^124^I
Thyroid	18%	17%	15%	19%	18%	18%
Liver	17%	17%	16%	19%	19%	19%
Kidneys	18%	17%	15%	19%	18%	18%
GI tract	16%	18%	17%	18%	77%	60%
Rem. body	18%	17%	15%	19%	18%	18%

## Data Availability

Not applicable.

## Appendix A

Here, we derive the Equation for uncertainty of cumulated activity ${\tilde{A}}_{5+}$ beyond the last measured time point ($t_{5}$ in this work) when extrapolating from biological washout $\lambda_{b}$. Exponential decay beyond $t_{5}$ is given by $A\left(t\right)=A_{5}e^{-\left(\lambda_{p}+\lambda_{b}\right)t}$ and the cumulated activity from $t_{5}$ to infinity is ${\tilde{A}}_{5+}=A_{5}/\left(\lambda_{p}+\lambda_{b}\right)$. Since $\lambda_{b}+\lambda_{p}=\ln\left(A_{4}/A_{5}\right)/\left(t_{5}-t_{4}\right)$ (Equation (2)), we have:

(A1) $${\tilde{A}}_{5+}=A_{5}\frac{t_{5}-t_{4}}{\ln\left(A_{4}/A_{5}\right)}$$

Using error propagation and assuming negligible uncertainty in the time points $t_{4}$ and $t_{5}$, as well as similar uncertainties $\delta A_{4}\approx \delta A_{5}$, the uncertainty for ${\tilde{A}}_{5+}$ becomes:

(A2) $$\begin{array}{c} \delta {\tilde{A}}_{5+}=\sqrt{{\left(\frac{\partial {\tilde{A}}_{5+}}{\partial A_{4}}\right)}^{2}{\left(\delta A_{4}\right)}^{2}+{\left(\frac{\partial {\tilde{A}}_{5+}}{\partial A_{5}}\right)}^{2}{\left(\delta A_{5}\right)}^{2}}\\ =\sqrt{{\left(\frac{-A_{5}\left(t_{5}-t_{4}\right)}{A_{4}{\left(\ln\left(A_{4}/A_{5}\right)\right)}^{2}}\right)}^{2}{\left(\delta A_{4}\right)}^{2}+{\left(\frac{\left(t_{5}-t_{4}\right)}{\ln\left(A_{4}/A_{5}\right)}+\frac{\left(t_{5}-t_{4}\right)}{{\left(\ln\left(A_{4}/A_{5}\right)\right)}^{2}}\right)}^{2}{\left(\delta A_{5}\right)}^{2}}\\ =\frac{\delta A_{5}}{A_{5}}\;{\tilde{A}}_{5+}\cdot \frac{1}{\ln\left(A_{4}/A_{5}\right)}\sqrt{{\left(\frac{A_{5}}{A_{4}}\right)}^{2}+{\left(1+\ln\left(\frac{A_{4}}{A_{5}}\right)\right)}^{2}} \end{array}$$

The relative uncertainty in cumulated activity ${\tilde{A}}_{5+}$ can then be expressed as a function of the measured activities $A_{4}$ and $A_{5}$ and the relative measurement uncertainty of the activity $A_{5}$:

(A3) $$\frac{\delta {\tilde{A}}_{5+}}{{\tilde{A}}_{5+}}=\frac{\delta A_{5}}{A_{5}}\;\cdot \;\eta \left(\frac{A_{4}}{A_{5}}\right)$$

where the factor η has been defined as and is graphed in Figure A1:

(A4) $$\eta \left(\frac{A_{4}}{A_{5}}\right)\;\equiv \;\frac{1}{\ln\left(A_{4}/A_{5}\right)}\sqrt{{\left(\frac{A_{5}}{A_{4}}\right)}^{2}+{\left(1+\ln\left(\frac{A_{4}}{A_{5}}\right)\right)}^{2}}$$

## References

[1] Yordanova A., Eppard E., Kürpig S., Bundschuh R.A., Schönberger S., Gonzalez-Carmona M., Feldmann G., Ahmadzadehfar H., Essler M. (2017). Theranostics in nuclear medicine practice. Oncol. Targets Ther..

[2] Langbein T., Weber W.A., Eiber M. (2019). Future of Theranostics: An Outlook on Precision Oncology in Nuclear Medicine. J. Nucl. Med..

[3] Goldsmith S.J. (2020). Targeted Radionuclide Therapy: A Historical and Personal Review. Semin. Nucl. Med..

[4] Becker D.V., Sawin C.T. (1996). Radioiodine and Thyroid Disease: The Beginning. Semin. Nucl. Med..

[5] National Nuclear Data Center Nuclear Decay Data in the MIRD Format. https://www.nndc.bnl.gov/mird/.

[6] Busk M., Overgaard J., Horsman M.R. (2020). Imaging of Tumor Hypoxia for Radiotherapy: Current Status and Future Directions. Semin. Nucl. Med..

[7] Wiebe L.I., McEwan A.J.B. (2002). Scintigraphic Imaging of Focal Hypoxic Tissue: Development and Clinical Applications of ^123^I-IAZA. Brazilian Arch. Biol. Technol..

[8] Stypinski D., McQuarrie S.A., McEwan A.J.B., Wiebe L.I. (2018). Pharmacokinetics and Scintigraphic Imaging of the Hypoxia-Imaging Agent [^123^I]IAZA in Healthy Adults Following Exercise-Based Cardiac Stress. Pharmaceutics.

[9] Stypinski D., McQuarrie S.A., Wiebe L.I., Tam Y.K., Mercer J.R., McEwan A.J.B. (2001). Dosimetry Estimations for [^123^I]IAZA in Healthy Volunteers. J. Nucl. Med..

[10] Loevinger R., Budinger T.F., Watson E.E. (1991). MIRD Primer for Absorbed Dose Calculations.

[11] Watson E.E., Stabin M.G., Siegel J.A. (1993). MIRD formulation. Med. Phys..

[12] Stabin M.G., Sparks R.B., Crowe E. (2005). OLINDA/EXM: The Second-Generation Personal Computer Software for Internal Dose Assessment in Nuclear Medicine. J. Nucl. Med..

[13] Thomas S.R., Stabin M.G., Chen C.T., Samaratunga R.C. (1999). MIRD Pamphlet No. 14 Revised: A Dynamic Urinary Bladder Model for Radiation Dose Calculations. J. Nucl. Med..

[14] International Commission on Radiological Protection (1979). Limits for Intakes of Radionuclides by Workers.

[15] Gear J.I., Maurice GCox M.G., Gustafsson J., Gleisner K.S., Murray I., Glatting G., Konijnenberg M., Flux G.D. (2018). EANM practical guidance on uncertainty analysis for molecular radiotherapy absorbed dose calculations. Eur. J. Nucl. Med. Mol. Imag..

[16] Flux G.D., Guy M.J., Beddows R., Pryor M., Flower M.A. (2002). Estimation and implications of random errors in whole-body dosimetry for targeted radionuclide therapy. Phys. Med. Biol..

[17] Eberlein U., Cremonesi M., Lassmann M. (2017). Individualized Dosimetry for Theranostics: Necessary, Nice to Have, or Counterproductive?. J. Nucl. Med..

[18] Smyth V. (2015). Metrology for Molecular Radiotherapy.

[19] Miller C., Rousseau J., Ramogida C.F., Celler A., Rahmim A., Uribe C.F. (2022). Implications of physics, chemistry and biology for dosimetry calculations using theranostic pairs. Theranostics.

